# Supplementary material for: COVID-IRS: A novel predictive score for risk of invasive mechanical ventilation in patients with COVID-19
Source: PLoS One. 2021 Apr 5;16(4):e0248357. doi: 10.1371/journal.pone.0248357 (PMC8021150; doi:10.1371/journal.pone.0248357)
Supplement: S1 Table — Values are percentages or median (IQR) as appropriate. IMV: Invasive Mechanical Ventilation, BMI: Body Mass Index, COPD: Chronic Obstructive Pulmonary Disease, CKD: Chronic Kidney Disease, SaO2: Oxygen saturation, FiO2: Fraction of inspired oxygen, NLR: Neutrophil/Lymphocyte Ratio, INR: International Normalized Ratio, AST: Aspartate Aminotransferase, ALT: Alanine Aminotransferase, ALP: Alkaline Phosphatase, GPT: Glutamic Pyruvic Transaminase, TB: Total Bilirubin, BUN: Blood Urea Nitrogen, CPK: Creatinine Phosphokinase, LDH: Lactate Dehydrogenase, IL-6: Interleukin 6, IgG: Immunoglobulin G, IgM Immunoglobulin M. (DOCX) [file pone.0248357.s001.docx]

**S1 Table. Comparison between the development and validation cohorts**

| **Supplementary Table 1. Comparison between the development and validation cohorts** | | | |
| --- | --- | --- | --- |
|  | **Development (n=211)** | **Validation (n=190)** | **p-value** |
| **Age, years** | 52.82 (44.09-65.72) | 55.19 (44.39-66.06) | 0.551 |
| **Male sex** | 149 (70.62) | 132 (69.47) | 0.803 |
| **BMI** | 27.78 (25.47-31.25) | 27.68 (24.80-31.25) | 0.419 |
| **Tabaquic index** | 3 (0.5-17.5) | 5.25 (1.6-20) | 0.198 |
| **Diabetes** | 40 (19.32) | 28 (14.97) | 0.254 |
| **Hypertension** | 62 (29.81) | 56 (29.95) | 0.976 |
| **COPD** | 3 (1.46) | 5 (2.69) | 0.393 |
| **CKD** | 3 (1.46) | 5 (2.69) | 0.393 |
| **Vital signs on admission** |  |  |  |
| Cardiac rate | 81 (75-90) | 80 (72-88) | 0.388 |
| Respiratory rate | 20 (18-24) | 20 (18-24) | 0.558 |
| Mean arterial pressure | 86.66 (80-92.33) | 86.67 (80-90.67) | 0.950 |
| Oxygen saturation | 90 (85-94) | 90 (85-94) | 0.852 |
| SaO2/FiO2 ratio | 217.5 (95-232) | 215 (99-232) | 0.937 |
| Temperature | 36.4 (36-36.8) | 36.4 (36-37) | 0.370 |
| **Days until admission*** | 8 (5-12) | 7 (6-11) | 0.630 |
| **Laboratory values** |  |  |  |
| Hemoglobin | 14.8 (13.4-16.1) | 14.8 (13.6-15.9) | 0.858 |
| Leucocytes | 7.4 (5.3-9.9) | 7.1 (5.4-9.7) | 0.900 |
| Lymphocytes | 1000 (710-1350) | 1005 (740-1460) | 0.392 |
| Neutrophils | 5345 (3560-7860) | 5390 (3850-8030) | 0.479 |
| NLR | 5.43 (3.10-9.20) | 5.37 (3.4-8.51) | 0.877 |
| Platelets | 216 (168-283) | 215 (168-278) | 0.776 |
| HbA1c | 5.9 (5.5-6.5) | 5.9 (5.5-6.4) | 0.786 |
| D-dimer | 842 (522-1403) | 844 (546-1211) | 0.453 |
| INR | 0.98 (0.92-1.06) | 0.99 (0.93-1.07) | 0.718 |
| Fibrinogen | 461 (363-570) | 414 (332-532) | 0.093 |
| Albumin | 3.77 (3.35-4.08) | 3.74 (3.36-4.06) | 0.744 |
| AST | 36.6 (23.8-55) | 37 (25.4-56) | 0.831 |
| ALT | 37 (22-57) | 32.5 (22-58) | 0.407 |
| AF | 80 (64-101) | 84 (64-108) | 0.289 |
| GGT | 88 (44-147) | 81 (42-137) | 0.625 |
| BT | 0.5 (0.35-0.7) | 0.52 (0.36-0.73) | 0.462 |
| Glucose | 116 (98.3-134) | 114.5 (98.85-134.95) | 0.851 |
| BUN | 14.25 (10.7-20.5) | 15.7 (11.7-20.1) | 0.353 |
| Creatinine | 0.89 (0.76-1.07) | 0.88 (0.73-1.07) | 0.441 |
| CPK | 112 (58-247.5) | 89 (51-186) | 0.050 |
| LDH | 287 (224-374) | 286 (219-373) | 0.774 |
| C Reactive Protein | 11.33 (4.09-21) | 11.08 (4.36-19) | 0.647 |
| Procalcitonin | 0.16 (0.08-0.43) | 0.14 (0.07-0.36) | 0.213 |
| Ferritin | 941 (460-1628) | 878 (405-1495) | 0.307 |
| IL-6 | 66.8 (28.4-146) | 61.45 (23.2-135) | 0.483 |
| IgG | 1150 (985-1331) | 1069 (920-1224) | 0.022 |
| IgM | 93 (62-132) | 97.7 (73-144.7) | 0.056 |
| **Death** | 13 (6.25) | 17 (8.95) | 0.309 |
| **Length of stay** | 9 (6-15) | 9 (6-20) | 0.329 |
| **COVID-19 treatment given** |  |  |  |
| Lopinavir/ritonavir | 127 (60.11) | 118 (61.94) | 0.734 |
| Azithromycin | 156 (74.01) | 146 (76.77) | 0.560 |
| Hydroxychloroquine | 173 (81.92) | 142 (74.68) | 0.109 |
| Tocilizumab | 90 (42.68) | 83 (43.54) | 0.879 |
| Corticosteroids | 162 (76.67) | 149 (78.61) | 0.099 |
| **Maximum ventilatory support used** | |  |  |
| Conventional nasal cannula | 61 (28.90) | 55 (28.94) | 0.993 |
| Face tent | 4 (1.89) | 1 (0.52) | 0.217 |
| Non-rebreather mask | 6 (2.84) | 10 (5.26) | 0.216 |
| BiPAP/CPAP | 7 (3.31) | 6 (3.15) | 0.928 |
| High flow nasal cannula | 21 (9.95) | 16 (8.42) | 0.597 |
| IMV | 72 (36.84) | 70 (34.12) | 0.570 |

Values are percentages or median (IQR) as appropriate. IMV: Invasive Mechanical Ventilation, BMI: Body Mass Index, COPD: Chronic Obstructive Pulmonary Disease, CKD: Chronic Kidney Disease, SaO2: Oxygen saturation, FiO2: Fraction of inspired oxygen, NLR: Neutrophil/Lymphocyte Ratio, INR: International Normalized Ratio, AST: Aspartate Aminotransferase, ALT: Alanine Aminotransferase, ALP: Alkaline Phosphatase, GPT: Glutamic Pyruvic Transaminase, TB: Total Bilirubin, BUN: Blood Urea Nitrogen, CPK: Creatinine Phosphokinase, LDH: Lactate Dehydrogenase, IL-6: Interleukin 6, IgG: Immunoglobulin G, IgM Immunoglobulin M. *Days from symptom onset until hospital admission
