# Supplementary material for: COVID-IRS: A novel predictive score for risk of invasive mechanical ventilation in patients with COVID-19
Source: PLoS One. 2021 Apr 5;16(4):e0248357. doi: 10.1371/journal.pone.0248357 (PMC8021150; doi:10.1371/journal.pone.0248357)
Supplement: S2 Table — BMI: Body Mass Index, COPD: Chronic Obstructive Pulmonary Disease, CKD: Chronic Kidney Disease, SaO2: Oxygen saturation, FiO2: Fraction of inspired oxygen, NLR: Neutrophil/Lymphocyte Ratio, INR: International Normalized Ratio, AST: Aspartate Aminotransferase, ALT: Alanine Aminotransferase, ALP: Alkaline Phosphatase, GPT: Glutamic Pyruvic Transaminase, TB: Total Bilirubin, BUN: Blood Urea Nitrogen, CPK: Creatinine Phosphokinase, LDH: Lactate Dehydrogenase, IL-6: Interleukin 6, IgG: Immunoglobulin G, IgM Immunoglobulin M. (DOCX) [file pone.0248357.s002.docx]

**S2 Table. Univariate logistic regressions for variable selection**

| **Variable** | **OR** | **p-value** | **95% CI** |
| --- | --- | --- | --- |
| Age | 1.014 | 0.113 | (0.996-1.033) |
| BMI | 1.044 | 0.091 | (0.993-1.098) |
| Tabaquic index | 0.986 | 0.377 | (0.956-1.017) |
| Male sex | 0.515 | 0.052 | (0.263-1.005) |
| Sistolic arterial pressure | 0.987 | 0.111 | (0.972-1.003) |
| Diastolic arterial pressure | 0.957 | 0.003 | (0.929-0.985) |
| Cardiac rate | 1.013 | 0.193 | (0.993-1.034) |
| Respiratory rate | 1.144 | <0.001 | (1.082-1.209) |
| Glasgow coma scale | 1.216 | 0.648 | (0.524-2.823) |
| Temperature | 0.857 | 0.266 | (0.653-1.124) |
| Oxygen saturation | 0.858 | <0.001 | (0.815-0.904) |
| SaO2/FiO2 ratio | 0.980 | <0.001 | (0.977-0.984) |
| COPD | 4.029 | 0.259 | (0.358-45.240) |
| Diabetes | 1.860 | 0.084 | (0.919-3.764) |
| Hypertension | 2.250 | 0.010 | (1.215-4.165) |
| Fever | 2.416 | 0.070 | (0.930-6.273) |
| Cough | 2.668 | 0.033 | (1.082-6.578) |
| Myalgias | 2.285 | 0.106 | (0.838-6.227) |
| Arthralgias | 2.081 | 0.144 | (0.778-5.564) |
| Headache | 0.866 | 0.746 | (0.362-2.067) |
| Anosmia | 1.163 | 0.784 | (0.395-3.423) |
| Disgeusia | 0.606 | 0.418 | (0.180-2.035) |
| Rhinorrea | 0.618 | 0.559 | (0.123-3.101) |
| Diarrhea | 1.068 | 0.882 | (0.446-2.554) |
| Breathlessness | 11.007 | 0.001 | (2.515-48.163) |
| Hemoglobin | 1.018 | 0.794 | (0.886-1.170) |
| Leucocytes | 1.161 | <0.001 | (1.079-1.249) |
| Neutrophils | 1.0001 | <0.001 | (1.0000-1.0002) |
| Lymphocytes | 0.9991 | 0.011 | (0.9985-0.9998) |
| NLR | 1.103 | <0.001 | (1.050-1.159) |
| Platelets | 0.999 | 0.716 | (0.996-1.002) |
| HbA1c | 1.507 | 0.017 | (1.075-2.112) |
| D Dimer | 1.0001 | 0.010 | (1.0000-1.0003) |
| INR | 7.939 | 0.398 | (0.0651-96.982) |
| Fibrinogen | 1.001 | 0.494 | (0.997-1.004) |
| Albumin | 0.177 | <0.001 | (0.0927-0.341) |
| AST | 1.010 | 0.007 | (1.002-1.017) |
| ALT | 1.009 | 0.017 | (1.001-1.017) |
| FA | 1.007 | 0.052 | (0.999-1.014) |
| GGT | 1.001 | 0.532 | (0.997-1.005) |
| BT | 3.607 | 0.003 | (1.550-8.392) |
| Glucose | 1.010 | <0.001 | (1.004-1.015) |
| Creatinine | 2.206 | 0.042 | (1.029-4.732) |
| BUN | 1.071 | <0.001 | (1.034-1.110) |
| CPK | 1.0001 | 0.096 | (0.999-1.001) |
| LDH | 1.007 | <0.001 | (1.004-1.0105) |
| Vitamin_D | 0.948 | 0.026 | (0.905-0.993) |
| C Reactive Protein | 1.087 | <0.001 | (1.055-1.119) |
| PCT | 3.289 | <0.001 | (1.862-5.810) |
| VSG | 1.006 | 0.775 | (0.963-1.050) |
| Ferritin | 1.0004 | 0.001 | (1.0001-1.0006) |
| IL-6 | 1.007 | <0.001 | (1.004-1.010) |
| IgG | 0.999 | 0.852 | (0.998-1.001) |
| IgM | 0.995 | 0.145 | (0.988-1.001) |

BMI: Body Mass Index, COPD: Chronic Obstructive Pulmonary Disease, CKD: Chronic Kidney Disease, SaO2: Oxygen saturation, FiO2: Fraction of inspired oxygen, NLR: Neutrophil/Lymphocyte Ratio, INR: International Normalized Ratio, AST: Aspartate Aminotransferase, ALT: Alanine Aminotransferase, ALP: Alkaline Phosphatase, GPT: Glutamic Pyruvic Transaminase, TB: Total Bilirubin, BUN: Blood Urea Nitrogen, CPK: Creatinine Phosphokinase, LDH: Lactate Dehydrogenase, IL-6: Interleukin 6, IgG: Immunoglobulin G, IgM Immunoglobulin M.
