# Supplementary material for: COVID-IRS: A novel predictive score for risk of invasive mechanical ventilation in patients with COVID-19
Source: PLoS One. 2021 Apr 5;16(4):e0248357. doi: 10.1371/journal.pone.0248357 (PMC8021150; doi:10.1371/journal.pone.0248357)
Supplement: S3 Table — SaO2: Oxygen saturation, FiO2: Fraction of inspired oxygen, LDH: Lactate Dehydrogenase, IL-6: Interleukin 6, NLR: Neutrophil/Lymphocyte Ratio. (DOCX) [file pone.0248357.s003.docx]

**S3 Table. Multivariate logistic regression**

| **Variable** | **Coefficient** | **Standard error** | **p-value** | **95% CI** | | **Pseudo R^2^** |
| --- | --- | --- | --- | --- | --- | --- |
| **COVID-IRS-NLR** | | | | | | |
| Respiratory rate | 0.069 | 0.025 | 0.006 | 0.0198 | 0.118 | 0.3428 |
| SaO2/FiO2 ratio | -0.013 | 0.002 | 0 | -0.017 | -0.008 |  |
| LDH | 0.004 | 0.001 | 0.001 | 0.001 | 0.006 |  |
| NLR | 0.049 | 0.017 | 0.004 | 0.016 | 0.083 |  |
| Constant | -1.763 | 0.921 | 0.056 | -3.569 | 0.043 |  |
| **COVID-IRS-IL6** | | | | | | |
| Respiratory rate | 0.060 | 0.025 | 0.017 | 0.010 | 0.110 | 0.3616 |
| SaO2/FiO2 ratio | -0.013 | 0.002 | 0 | -0.018 | -0.009 |  |
| LDH | 0.003 | 0.001 | 0.018 | 0.0005 | 0.0057 |  |
| IL-6 | 0.004 | 0.001 | 0 | 0.001 | 0.006 |  |
| Constant | -1.207 | 0.904 | 0.182 | -2.980 | 0.566 |  |

SaO2: Oxygen saturation, FiO2: Fraction of inspired oxygen, LDH: Lactate Dehydrogenase, IL-6: Interleukin 6, NLR: Neutrophil/Lymphocyte Ratio
