# Supplementary material for: COVID-IRS: A novel predictive score for risk of invasive mechanical ventilation in patients with COVID-19
Source: PLoS One. 2021 Apr 5;16(4):e0248357. doi: 10.1371/journal.pone.0248357 (PMC8021150; doi:10.1371/journal.pone.0248357)
Supplement: S4 Table — NLR: Neutrophil/Lymphocyte Ratio. (DOCX) [file pone.0248357.s004.docx]

**S4 Table. Spearman’s correlation results and R-squared of multivariate logistic regression models for surrogate variables**

| **Variable** | **Spearman’s correlation** | | **Multivariate logistic regression** | | | | |
| --- | --- | --- | --- | --- | --- | --- | --- |
|  | **Rho** | **p-value** | **Coefficient** | **p-value** | **95% CI** | | **Pseudo R^2^** |
| Leucocytes | 0.431 | <0.001 | 0.054 | 0.174 | -0.024 | 0.133 | 0.3570 |
| Neutrophils | 0.453 | <0.001 | 0.00006 | 0.110 | -0.00001 | 0.0001 | 0.3554 |
| Lymphocytes | -0.277 | <0.001 | -0.0005 | 0.111 | -0.001 | 0.0001 | 0.3588 |
| NLR | 0.485 | <0.001 | 0.049 | 0.004 | 0.016 | 0.083 | 0.3428 |
| Platelets | -0.030 | 0.576 | -0.002 | 0.075 | -0.005 | 0.0002 | 0.3571 |
| D Dimer | 0.298 | <0.001 | -0.00006 | 0.447 | -0.0002 | 0.00009 | 0.3539 |
| C Reactive Protein | 0.660 | <0.001 | -0.0001 | 0.956 | -0.006 | 0.005 | 0.3526 |
| Procalcitonin | 0.556 | <0.001 | 0.055 | 0.343 | -0.059 | 0.171 | 0.3496 |
| Ferritin | 0.236 | <0.001 | 4.88e-06 | 0.958 | -0.0001 | 0.0001 | 0.3490 |

NLR: Neutrophil/Lymphocyte Ratio
