# Supplementary figures and images for: COVID-IRS: A novel predictive score for risk of invasive mechanical ventilation in patients with COVID-19
Source: PLoS One. 2021 Apr 5;16(4):e0248357. doi: 10.1371/journal.pone.0248357 (PMC8021150; doi:10.1371/journal.pone.0248357)

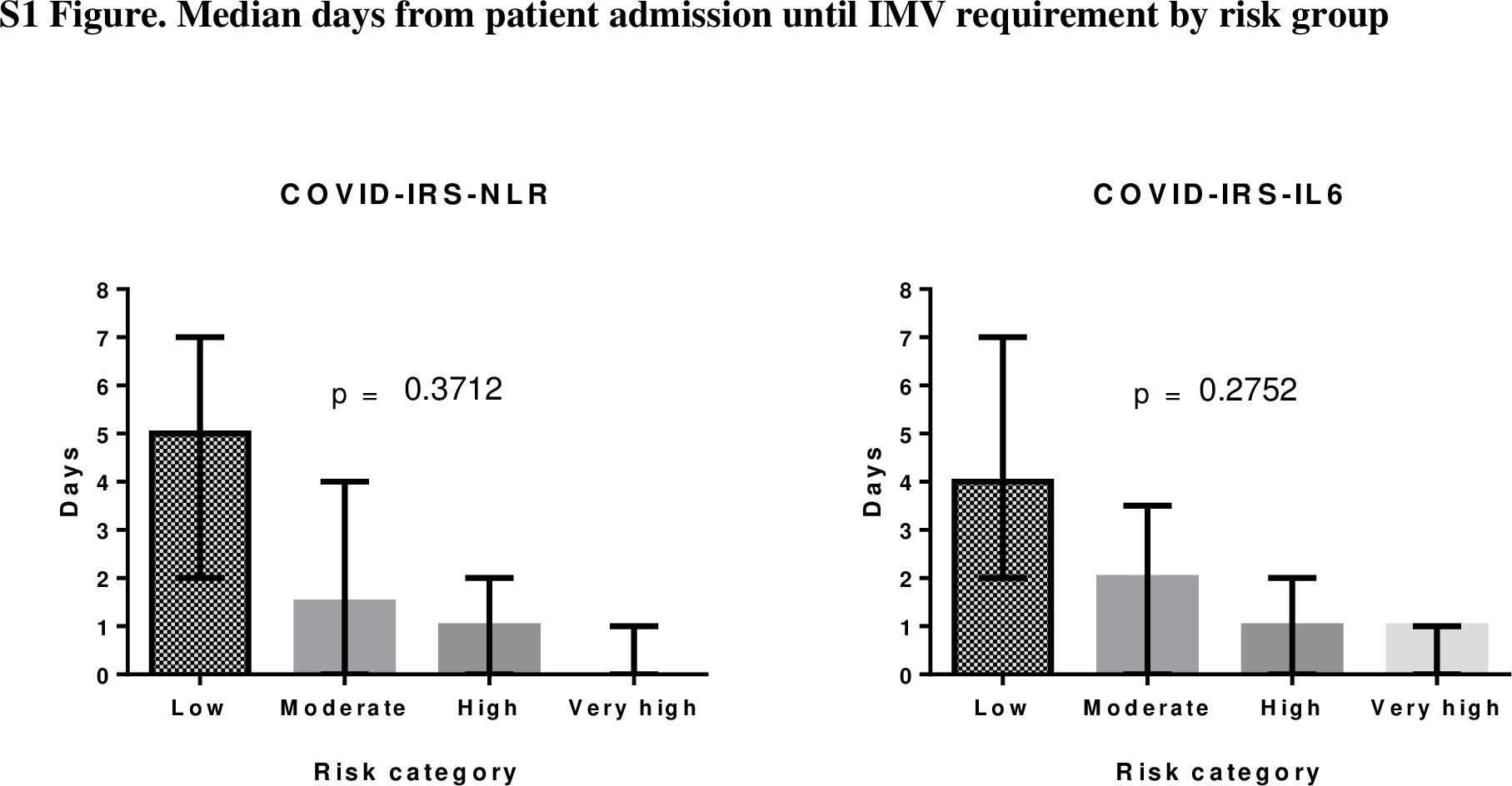

Supplement: S1 Fig — Here we show the median time in days from patient admission until the patients required the initiation of IMV. There was a tendency towards a higher median amount of days between patient admission to the hospital and the requirement of IMV in lower risk groups. These differences did not prove to be statistically significant (COVID-IRS-NLR, p = 0.371; COVID-IRS-IL6, p = 0.275). (TIF) [file pone.0248357.s005.tif]

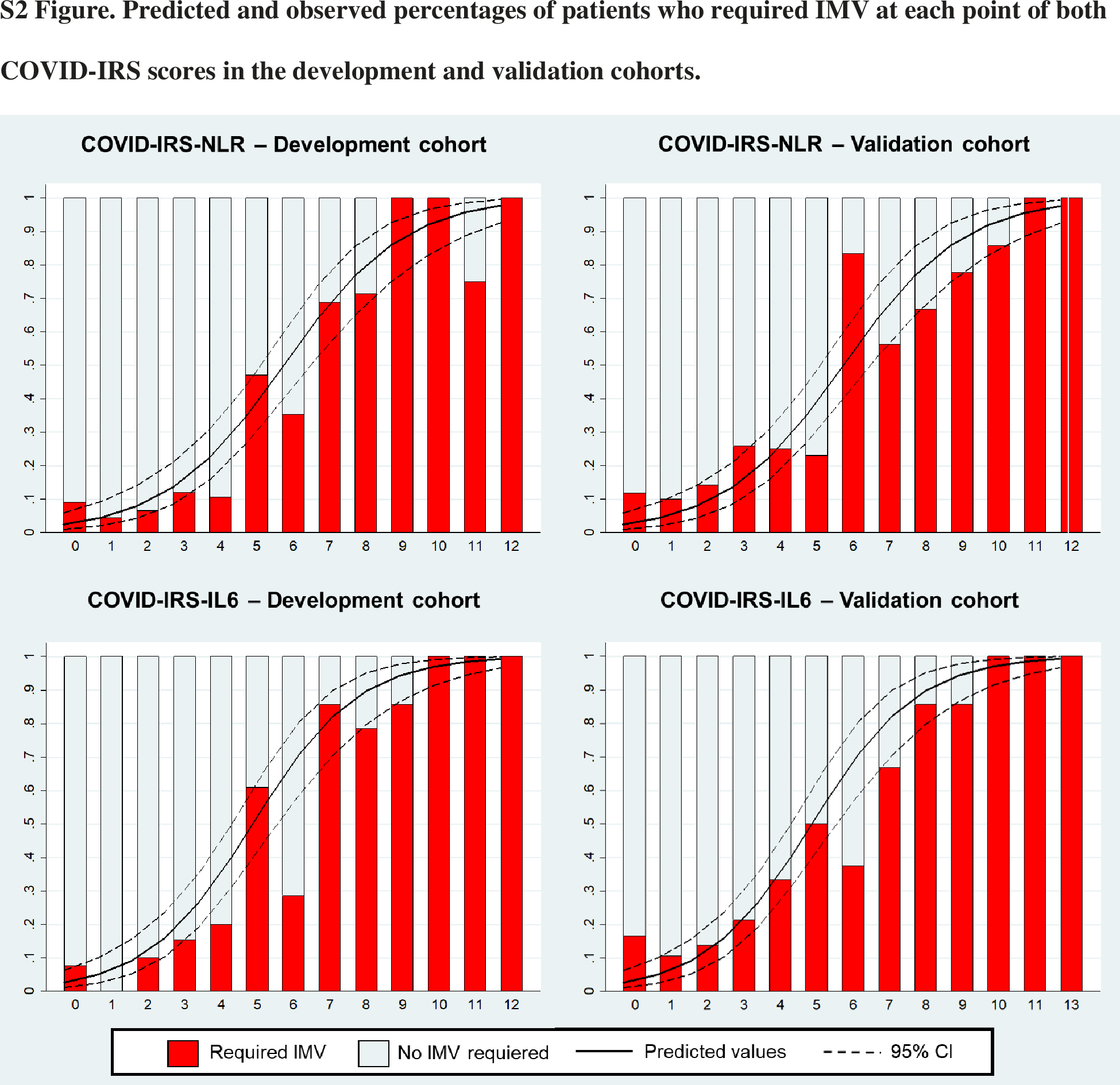

Supplement: S2 Fig — Here we show the correlation between observed and predicted percentages of patients who required IMV. Both predicted and measured risks showed a strong correlation. (TIF) [file pone.0248357.s006.tif]
