# Supplementary material for: COVID-IRS: A novel predictive score for risk of invasive mechanical ventilation in patients with COVID-19
Source: PLoS One. 2021 Apr 5;16(4):e0248357. doi: 10.1371/journal.pone.0248357 (PMC8021150; doi:10.1371/journal.pone.0248357)
Supplement: Attachment — Submitted filename: Poin by point rebuttal COVID-IRS Plos One rev 210126.docx [file pone.0248357.s007.docx]

January 26, 2021

Dr. Antonio Palazón-Bru
Academic Editor
PLOS ONE

Dear Professor Palazón-Bru,

Along with this letter you will find the revised version of our manuscript entitled ***COVID-IRS: a novel predictive score for risk of invasive mechanical ventilation in patients with COVID-19*** (manuscript number PONE-D-20-32632). We have carefully read the comments and observations made by the reviewers and have answered them point-by-point, as per the document attached to this letter. As instructed, all modifications made to the original manuscript were made with track changes. We reiterate that all authors have significantly contributed to this work, be it in the conception and design of the study, the acquisition of data, the analysis and interpretation of the results and the preparation of the manuscript. We would also like to take this opportunity to thank you and your reviewers for your valuable contribution to improve the quality of our manuscript.

Respectfully,

Antonio Camiro Zúñiga, MD

Corresponding author

Journal Requirements:

When submitting your revision, we need you to address these additional requirements.

1. Please ensure that your manuscript meets PLOS ONE's style requirements, including those for file naming. The PLOS ONE style templates can be found at

<https://journals.plos.org/plosone/s/file?id=wjVg/PLOSOne_formatting_sample_main_body.pdf> and

<https://journals.plos.org/plosone/s/file?id=ba62/PLOSOne_formatting_sample_title_authors_affiliations.pdf>

Thank you for this observation, the manuscript was edited according to the format required by PLOS ONE.

2.Thank you for including your ethics statement: 'The protocol was approved by our scientific and ethics committees and conducted according to the principles of the Helsinki declaration.'

(a) Please amend your current ethics statement to include the full name of the ethics committee/institutional review board(s) that approved your specific study.
(b) Once you have amended this/these statement(s) in the Methods section of the manuscript, please add the same text to the “Ethics Statement” field of the submission form (via “Edit Submission”).

The protocol (protocol ID ABC-20-50) was approved by our local investigation and ethics (*Comité de Ética en Investigación, Centro Médico ABC*) and conducted according to the principles of the Helsinki declaration, this information was added to the manuscript and to the submission platform.

For additional information about PLOS ONE ethical requirements for human subjects research, please refer to <http://journals.plos.org/plosone/s/submission-guidelines#loc-human-subjects-research>.

3. In ethics statement in the manuscript and in the online submission form, please provide additional information about the patient records/samples used in your retrospective study. Specifically, please ensure that you have discussed whether all data/samples were fully anonymized before you accessed them and/or whether the IRB or ethics committee waived the requirement for informed consent. If patients provided informed written consent to have data/samples from their medical records used in research, please include this information.

The ethics committee waived the requirement for an informed consent. All the analyzed data was fully anonymized during the capturing process and was fully anonymized before access and analysis. This information was included in the manuscript.

4. Please note that PLOS does not permit references to “data not shown.” Authors should provide the relevant data within the manuscript, the Supporting Information files, or in a public repository. If the data are not a core part of the research study being presented, we ask that authors remove any references to these data.

Regarding the relevant data missing, the data has been added as a supplementary figure S5.

5. Please amend your list of authors on the manuscript to ensure that each author is linked to an affiliation. Authors’ affiliations should reflect the institution where the work was done (if authors moved subsequently, you can also list the new affiliation stating “current affiliation:….” as necessary).

Affiliations have been revised and this has been corrected in the manuscript.

6. One of the noted authors is a group or consortium [ARMII Study Group]. In addition to naming the author group, please list the individual authors and affiliations within this group in the acknowledgments section of your manuscript. Please also indicate clearly a lead author for this group along with a contact email address.

Mercedes Aguilar Soto is the lead author of the ARMII Study Group, all the authors are affiliated to Centro Médico ABC in México City. This information has been added to the manuscript.

7. Please amend either the abstract on the online submission form (via Edit Submission) or the abstract in the manuscript so that they are identical.

[Note: HTML markup is below. Please do not edit.]

Reviewers' comments:

Reviewer's Responses to Questions

**Comments to the Author**

1. Is the manuscript technically sound, and do the data support the conclusions?

The manuscript must describe a technically sound piece of scientific research with data that supports the conclusions. Experiments must have been conducted rigorously, with appropriate controls, replication, and sample sizes. The conclusions must be drawn appropriately based on the data presented.

Reviewer #1: Yes

Reviewer #2: Yes

2. Has the statistical analysis been performed appropriately and rigorously?

Reviewer #1: N/A

Reviewer #2: Yes

3. Have the authors made all data underlying the findings in their manuscript fully available?

The [PLOS Data policy](http://www.plosone.org/static/policies.action#sharing) requires authors to make all data underlying the findings described in their manuscript fully available without restriction, with rare exception (please refer to the Data Availability Statement in the manuscript PDF file). The data should be provided as part of the manuscript or its supporting information, or deposited to a public repository. For example, in addition to summary statistics, the data points behind means, medians and variance measures should be available. If there are restrictions on publicly sharing data—e.g. participant privacy or use of data from a third party—those must be specified.

Reviewer #1: No

Reviewer #2: Yes

4. Is the manuscript presented in an intelligible fashion and written in standard English?

PLOS ONE does not copyedit accepted manuscripts, so the language in submitted articles must be clear, correct, and unambiguous. Any typographical or grammatical errors should be corrected at revision, so please note any specific errors here.

Reviewer #1: Yes

Reviewer #2: Yes

5. Review Comments to the Author

Please use the space provided to explain your answers to the questions above. You may also include additional comments for the author, including concerns about dual publication, research ethics, or publication ethics. (Please upload your review as an attachment if it exceeds 20,000 characters)

Reviewer #1: Garcia-Gordillo conducted a retrospective study to investigate the new models to predict the need of IMV in patients with COVID-19. Although both NLR and IL-6 were well recognized as indicators of worse outcome in patients with covid-19, this study provided some useful information for clinical practices. I have some comments:

1) It’s problematic to use variables in the model at your own discretion which you describe in the method section. These variables should be determined base on standard statistic methods.

- Response: Using the development cohort, we performed univariate logistic regressions for IMV using all the variables mentioned above. We selected all variables that had a p value <0.1 and conducted a backwards stepwise multivariate logistic regression to find the variables that were independently associated with the requirement of IMV. After the selection of the optimal variables for the model, in order to ensure the model’s applicability in most settings, we checked for the variable’s availability in general settings. This was done by checking on 7 different general hospitals in Mexico City and its surroundings. The variables that were not available in more than 50% of the screened hospitals were deemed to be not readily available. We tested for similar variables using the Spearman correlation test in order to identify suitable surrogates. Thus, we developed two predictive models, one constructed with optimal variables and the other one with accessible surrogate variables. This information was included in the method section.

2) The authors described in the abstract that “When compared with other similar 42 scores developed for the prediction of adverse outcomes in COVID-19, the COVID-IRS 43 scores proved to be superior in the prediction of IMV.” However, no comparisons were done and no p values were given in Table 2. Moreover, more scores such as APACHE-Ⅱand SOFA should be compared with the models.

- Response: Thank you for your observation, ROC curves comparisons have been added to Table 2. Even though comparisons with APACHE-II would be very valuable for our analysis, the EMR does not include all the variables required for the calculation of this score. We have calculated SOFA score and compared it with our score, nevertheless SOFA score includes a variable for IMV so scores are overestimated. In addition to this, due to the retrospective nature of our data, we did not distinguish patients who needed IMV on arrival from those who were intubated through their hospital stay. This was included in the manuscript.

3) The optimal cutoff value of these models should be shown in order to provide more information for clinical practices.

- Response: Optimal cutoff points in the validation cohort for the COVID-IRS-NLR score and the COVID-IRS-IL6 were >6 (S: 68.57%, E: 87.5%) and >5 (S: 72.86%, E: 81.67%), respectively. This information has been added to the manuscript.

4) This study needs to be reviewed by a statistician.

- Response: The study was reviewed by a statistician as suggested by the reviewer

Reviewer #2: I am pleased to read a good work such this even if retrospective. I think some clarifications are needed in order to be published and in my opinion are minor revisions.

1) All the patients were evaluated to be, in case of need, "resuscitated"? I mean, It's important to clarify there was not a DNR order for some patients.

- Response: Thank you for your comment, none of the patients included in our study had a DNR order at the time of admission or during hospitalization. This information was included in the manuscript.

2) If possible, in order to identify the inflammatory phase, I would put the variable "time since symptoms onset to ED admission".

- Response: We appreciate the observation. In the development cohort the time from symptom onset to hospital admission was a median from 8 days (IQR 5.12) while in the validation cohort the median days from symptom onset to admission was of 7 days (IQR 6-11) (p= 0.63). This has been added to table 1.

3) All the patients came from community? Nosocomial or health care related infections?

- Response: SARS-Cov-2 infection was community acquired in all of our patients.

4) I would explain which treatment (corticosteroids, remdesivir, immunomodulators) have been given to the patients belonging to the 2 groups.

- Response: Thank you for this observation, several treatments were prescribed to the patients according to current guidelines, with no difference between groups. Lopinavir/ritonavir was given to 61.94% vs 60.11% of patients in the validation and development cohorts respectively (p=0.734); azithromycin was given to 76.77% vs 74.01% of patients in the validation and development cohorts respectively (p=0.560); hydroxychloroquine was given to 74.68% vs 81.92% of patients in the validation and development cohorts respectively (p=0.109); tocilizumab was given to 43.54% vs 42.68% of patients in the validation and development cohorts respectively (p=0.879); high dose glucocorticoids was given to 78.61% vs 76.67% of patients in the validation and development cohorts respectively (p=0.099). Remdesivir was not available in Mexico during our study period. This information was included in the manuscript and table 1.

5) If not intubated, I would explain which kind of ventilatory support was given (nasal flow cannula, venturimask, CPAP, BiPAP)

- Response: Regarding the use of oxygen therapy in patients who did not require IMV, 65.2% in the development cohort and 69.2% in the validation cohort were treated with conventional nasal cannula (p=0.579). Three percent of the patients in the development cohort and none in the validation cohort required face tent (p=0.108). For non-rebreather mask the percentages were 8.97% for the development and 6.52 for the validation cohort (p=0.549). High-flow nasal cannula was required by 15% of the patients in the development cohort and 15.11% in the validation cohort (p=0.981). BIPAP/CPAP was used in 2.5% of the patients in the development cohort and 5.04% in the validation cohort (p=0.291). This information has been added to the manuscript.

6) I woud cite in the discussion the Brescia-COVID Respiratory Severity Scale as the first score that was made worldwide out of China (Brescia has been, with Bergamo, the epicenter in Italy and still the deadliest place in Europe). The PREDICO score (multicenter study from Bologna-Italy) 10.1016/j.cmi.2020.08.003 should be take into consideration.

- Response: Thank you for this observation. Information regarding this scores has been added to the discussion and the PREDI-CO score was applied to our population and had a AUC of 0.704..

7) I am surprised the C-reactive protein did not find a place into the multivariate analysis, how can you explain instead the choice of N/L ratio?

- Response: The C-reactive protein was strongly correlated with the need for IMV in the univariate analysis, however, in the multivariate analysis, both the IL-6 and the NLR consistently outperformed the CRP’s predictive value, which was also a surprise for us.
